# Immune suppression by myeloid-derived suppressor cells, MDSCs, in MYCN-driven neuroblastoma provides a potential target for cancer immunotherapy

**DOI:** 10.1186/2051-1426-2-S3-P203

**Published:** 2014-11-06

**Authors:** Nina Eissler, Baldur Sveinbjörnsson, Anna Kock, John Inge Johnsen, Per Kogner

**Affiliations:** 1 Karolinska Institutet, Stockholm, Sweden; 2 University of Tromsö, Tromsö, Norway

## Background

Novel developments in tumor-immunotherapy show promising results in cancer therapy and might provide new opportunities for neuroblastoma treatment. To define suitable immunological targets, it is crucial to understand the role of the immune system and the mechanisms underlying neuroblastoma-induced immune suppression. We previously demonstrated accumulation of pro-inflammatory immune cells within tumor tissues and effective anti-inflammatory treatment with low-dose aspirin in the TH-MYCN mouse model. This model can be further used to dissect detailed mechanisms of neuroblastoma-induced immune suppression relevant for tumor development and tumor progression with the overall aim to define suitable targets for neuroblastoma immunotherapy.

## Methods

Flow-cytometric analysis of tumor tissues and spleens were performed, as well as *in vitro *coculture assays with splenocytes of neuroblastoma-bearing TH-MYCN mice. Immune-cell status and functions were compared to wildtype controls. Reagents targeting immune suppressive mechanisms were tested *in vitro*. Coculture-assays using tumor supernatant-treated bone marrow cells were established to define factors responsible for the induction of suppressive immune cells.

## Results

Neuroblastoma-bearing animals develop splenomegaly, indicating an active role of the peripheral immune system during MYCN-driven neuroblastoma development. Flow-cytometric analysis of spleens showed accumulation of CD11b^+^Ly6c^+^Ly6g^+ ^myeloid-derived suppressor cells (MDSC) in tumor-bearing mice compared to controls. The myeloid cell populations found in tumor-bearing animals expressed high levels of PD1-binding ligand, PD-L1, suggesting a mechanism of T-cell inhibition and a possible target for immunotherapy. In spleens, total T cell numbers were reduced, while the CD4/CD8 ratio and expression of activation markers remained unchanged. RegulatoryT cells were present in tumor tissues, but their frequency was not enhanced in spleens of cancer-bearing animals, indicating that MDSCs play a major role in peripheral immune suppression and tumor progression. Sorted from spleens of tumor-bearing mice, these MDSCs were capable of suppressing T cell proliferation *in vitro *through the production of indolamine-2,3-dioxygenase (IDO) and inducible nictric oxide synthase (iNOS). To dissect the mechanisms of MDSC-induction in neuroblastoma, naïve bone marrow cells were cultured with neuroblastoma tumor cell line supernatants. This led to the development of suppressive cells with an "MDSC-like" phenotype. This system will be further exploited to define key factors enhancing the accumulation of MDSCs in this malignancy.

## Conclusions

Tumor-promoting inflammation contributes to neuroblastoma tumor development with active involvement of the peripheral immune system. Further studies are needed to define factors of the tumor microenvironment inducing direct or indirect immune-suppression. Immunological interventions by targeting various suppressive mechanisms will be evaluated aiming at future clinical therapeutic application.

